# Extracellular Lactate: A Novel Measure of T Cell Proliferation

**DOI:** 10.4049/jimmunol.1700886

**Published:** 2017-12-29

**Authors:** James T. Grist, Lorna B. Jarvis, Zoya Georgieva, Sara Thompson, Harpreet Kaur Sandhu, Keith Burling, Ashley Clarke, Sarah Jackson, Mark Wills, Ferdia A. Gallagher, Joanne L. Jones

**Affiliations:** * Department of Radiology, School of Clinical Medicine, University of Cambridge, Cambridge CB2 0QQ, United Kingdom;; † Department of Clinical Neurosciences, School of Clinical Medicine, University of Cambridge, Cambridge CB2 0AH, United Kingdom;; ‡ Core Biochemical Assay Laboratory, School of Clinical Medicine, University of Cambridge, Cambridge CB2 0QQ, United Kingdom; and; § Department of Medicine, School of Clinical Medicine, University of Cambridge, Cambridge CB2 0QQ, United Kingdom

## Abstract

Following activation, T cells rapidly divide and acquire effector functions. This energetically demanding process depends upon the ability of T cells to undergo metabolic remodeling from oxidative phosphorylation to aerobic glycolysis, during which glucose is converted into lactate and released extracellularly. In this article, we demonstrate that extracellular lactate can be used to dynamically assess human T cell responses in vitro. Extracellular lactate levels strongly correlated with T cell proliferation, and measuring lactate compared favorably with traditional methods for determining T cell responses (i.e., [^3^H]thymidine incorporation and the use of cell proliferation dyes). Furthermore, we demonstrate the usefulness of measuring lactate as a read-out in conventional suppression assays and high-throughput peptide-screening assays. Extracellular lactate was stably produced over 7 d, and results were reproducibly performed over several freeze–thaw cycles. We conclude that the use of extracellular lactate measurements can be a sensitive, safe, stable, and easy-to-implement research tool for measuring T cell responses and cellular metabolic changes in vitro.

## Introduction

T cells have complex environment-dependent metabolic profiles, with different T cell subsets using different metabolic pathways to fuel their energy requirements (1, 2). For example, in the presence of oxygen, resting naive and memory effector T cells (Teffs) primarily metabolize glucose to pyruvate, which then enters the mitochondrial TCA cycle to produce NADH, which acts as an electron donor for the electron transport chain, fueling ATP production via oxidative phosphorylation (OXPHOS) (2, 3). However, upon stimulation through the TCR and in the presence of CD28 costimulation, Teffs rapidly switch from OXPHOS to glycolysis to meet the increased energy and biosynthesis demands of cellular activation and proliferation (4, 5). This shift causes an increase in the production and subsequent excretion of lactate by the cell, which is formed in the cytosol by the action of lactate dehydrogenase. The upregulation of glycolytic metabolism in Teffs upon stimulation (even in the presence of oxygen) is akin to the “Warburg effect” observed in oncological cell lines, through which cancerous cells increase their lactate output with respect to healthy tissue to fuel their ever-increasing metabolic demands for rapid cellular proliferation and expansion (4, 6, 7). In contrast to Teffs, in normoxic conditions, resting regulatory T cells (Tregs) use fatty acids, rather than glucose, as their primary energy source, and they do not switch their metabolism from OXPHOS to aerobic glycolysis following in vitro TCR/CD28 stimulation (8–10).

Given the reliance of activated Teffs on Warburg metabolism, we set out to explore the usefulness of quantifying extracellular lactate as a measure of Teff proliferation, under standard laboratory normoxic conditions. In this article, we show that extracellular lactate compares favorably with more traditional measures of T cell proliferation (i.e., thymidine DNA incorporation and cell proliferation dye dilution assessed by flow cytometry). Because naturally occurring Tregs do not increase their production of lactate in response to CD3/CD28 stimulation in vitro, we demonstrate the usefulness of measuring lactate as a read-out of Treg-mediated suppression of Teff proliferation. Finally, given the stability of lactate and the speed and ease with which it can be measured, we demonstrate the potential of using it in T cell–screening assays (e.g., as a read-out of CMV exposure status) (11).

## Materials and Methods

### Cell preparation

Human PBMCs were isolated from the whole blood of healthy donors by Ficoll centrifugation (Amersham Pharmacia Biotech). All individuals gave written consent, and the study was approved by a local ethical review committee (REC: 11/EE/0007). PBMCs were immediately suspended in culture medium (RPMI 1640; Life Technologies) containing 1% penicillin, 1% streptomycin, and 10% FCS (S5394; Sigma-Aldrich) and adjusted to a concentration of 10^6^ viable cells per milliliter for subsequent assays. Pan T cells were separated magnetically (Pan T Cell Isolation Kit, II; Miltenyi Biotec), according to the manufacturer’s instructions. CD4^+^CD25^−^ and CD8^+^CD25^−^ Teffs and CD4^+^CD127^low^CD25^hi^ Tregs were isolated from Pan T cells by FACS (BD Influx), following staining of cell surface CD4, CD8, CD25, and CD127 with relevant Abs (eBioscience, BD, and BioLegend).

For the CMV study, healthy seropositive and negative donors were recruited from the National Institutes of Health Research Cambridge BioResource (HBREC.2014.07). PBMCs were isolated using Lymphoprep (Axis-Shield, Oslo, Norway) density gradient centrifugation, and the samples were frozen in 10% DMSO (Sigma-Aldrich) and 90% FBS (Life Technologies, Thermo Fisher Scientific). Cryopreserved PBMCs were resuscitated before use in prewarmed DMEM (Sigma-Aldrich) in the presence of 10 U/ml Benzonase Nuclease (Millipore), followed by a 1-h incubation in warmed X-VIVO 15 medium (Lonza) supplemented with Benzonase Nuclease at 37°C. The cells were rested overnight at 37°C in X-VIVO 15 medium or RPMI 1640 (Sigma-Aldrich) supplemented with penicillin, streptomycin, and 10% FBS. CMV serostatus of all donors was confirmed by serological assessment of CMV IgG levels using a Captia Cytomegalovirus (CMV) IgG EIA test (Trinity Biotech), following the manufacturer’s instructions.

### Proliferation assays

CD4^+^ and CD8^+^ T cells were cultured in normoxic conditions at 37°C with 5% CO_2_, with and without anti-CD3/CD28 stimulation at a cell/bead ratio of 4:1. At various time points (as indicated in the text), supernatant was collected and frozen at −20°C for lactate measurement, and the number of viable cells at the end of culture was counted using a hemocytometer. To correlate lactate with proliferation and/or activation, 10^6^ CD4^+^ cells labeled with cell proliferation dye were cultured with and without anti-CD3 stimulation for 6 d. At days 1, 4, and 6, the supernatant was collected for lactate measurements, and cells were counterstained with live/dead exclusion dye (Zombie NIR; BioLegend) and anti-CD25 and anti-CD69 Abs (BD) and analyzed by flow cytometry. Lactate measurements were compared with output for cell proliferation (cell proliferation dye analysis by flow cytometry; cell proliferation dye high cells were marked as nonproliferating, and cell proliferation dye low cells were marked as proliferating). To assess the sensitivity of lactate measurements compared with thymidine incorporation, human PBMCs were cultured at 10^5^–1.5 × 10^3^ cells per well in triplicates, with and without stimulation using 1 μg/ml plate bound anti-CD3/soluble CD28. Thymidine (25 miC/ml) was added during the last 18 h of culture, and plates were pulsed on a Becton Coulter cell harvester at days 3 and 5.

### Lactate measurements

Media and supernatant lactate were measured spectrophotometrically using a Dimension EXL autoanalyzer (Siemens) with Flex reagent cartridges (product code DF16). In brief, lactate dehydrogenase, NAD, dihydrazine sulfate, and Tris buffer (40 U and 10, 180, and 100 mmol/l, respectively) were added to cell-free supernatants. The subsequent exchange of lactate to pyruvate, captured by the hydrazine compound, is directly proportional to the change in NAD^+^ to NADH^+^ (NADH) concentration measured at 340–383 nm, from which the initial lactate pool size was inferred. Lactate was not measurable in RPMI 1640 and 10% FBS alone. To assess the freeze–thaw stability of lactate, 3 × 10^6^ Pan T cells were cultured in three separate 2-ml wells. All supernatant was removed from the wells at day 2, and three 50-μl fresh supernatant samples from each well were analyzed for extracellular lactate. Subsequently, supernatants were also collected for analysis after one, two, and three freeze–thaw cycles, with samples left in the freezer for ≥24 h before thawing.

### Treg suppression assay

Teffs (CD4^+^CD25^−^ T cells) and Tregs were sorted using an Influx cell sorter (BD), according to their expression of CD4, CD25, and CD127. Cells were stained with Abs (BD) and sorted as follows: CD3^+^CD4^+^CD25^hi^CD127^low^ cells were considered Tregs, and CD3^+^CD4^+^CD25^−^ cells were considered CD4^+^ Teffs. To discriminate between Teffs and Tregs in the final analysis, Teffs were labeled with 5 μM cell proliferation dye V450, and Tregs were labeled with 5 μM cell proliferation dye V670 (both from eBioscience). Teffs were plated at 10^4^ cells per well in triplicate, with and without Tregs, in RPMI 1640 + 5% human AB serum. For the final analysis, dead cells were excluded using dead cell exclusion staining (Zombie NIR; eBioscience), and cells were counterstained with Abs to CD4. Tregs were titrated in doubling dilutions so that the Treg/Teff ratio was 1:1 to 1:16. Triplicate control wells of CD4^+^ T cells without stimulus, and Tregs, at various dilutions, with and without stimulus, were also cultured. Treg Suppression Inspector beads (Miltenyi Biotec) were used to stimulate the assay, according to the manufacturer’s instructions.

Supernatant was removed for lactate analysis, and the cells were analyzed by flow cytometry at day 6. A lactate suppression index, derived from lactate and flow results, was calculated using Eq. 1 and 2.

$$Lactate\;Suppression\;Index=\;\;\frac{T_{S}-(T_{Sup}-T_{Reg,\;Stim})}{T_{S}}$$ (1)

Eq. 1 shows the suppression index calculated from the extracellular lactate measurements, where *T_S_* is the lactate concentration in a stimulated well of 10^4^ Teffs alone (i.e., Treg/Teff ratio of 0:1), *T_Sup_* is the lactate concentration with a Treg/Teff ratio of 1:n, where *n* > 0, and *T_Ref,Stim_* is the lactate concentration from the 1:0 well (i.e., 10^4^ stimulated Tregs alone). The lactate concentration from the 1:0 well was selected because our preliminary data demonstrated that the lactate suppression index was not significantly altered by correcting for the exact number of Tregs in any given well (Supplemental Fig. 2), it would be impractical for most investigators to set up multiple Treg control wells, and most investigators report the 1:1 suppression index (showing the other ratios to demonstrate that suppression can be diluted out).

For analysis of suppression by flow cytometry, live Teffs were gated (see Fig. 5 for analysis strategy). The flow cytometry suppression index was calculated by taking the ratio between the proliferating and nonproliferating populations, as defined by Eq. 2.

$$Flow\;Cytometric\;Suppression\;Index=\;\frac{T_{Stim}^{V670\;Low}-T_{Sup}^{V670\;Low}}{T_{Stim}^{V670\;Low}}$$, (2)

where $T_{Stim}^{V670\;Low}$ is the proportion of proliferating V670^low^ Teffs in the stimulated culture, and $T_{Sup}^{V670\;Low}$ is the proportion of proliferating V670^low^ Teffs in the stimulated culture with Tregs. The suppression index for each well was calculated and averaged for each cell set.

### CMV peptide response assay

A total of 10^6^ PBMCs from 10 healthy donors were labeled with Cell Proliferation Dye eFluor 450 (eBioscience) and cultured at 10^5^ cells per well in triplicate, alone, with soluble anti-CD3 (1 μg/ml), or with two overlapping peptide pools of CMV immunodominant epitopes (IE1 and gB; 1 μg/ml). The peptide pools were consecutive 15mer peptides overlapping by 10 aa (libraries synthesized by ProImmune PEPscreen from previously published sequences) (11, 12). Five donors were naive to CMV, and five had known previous exposure. Donor status was assessed using CMV serology, as previously described. Cells were cultured in a 96-well plate for 6 d. At days 2, 5, and 6, triplicate supernatants were removed from the plate for lactate analysis. At the final time point (day 6), the cells were collected for flow cytometric analysis. Total lactate responses were corrected by subtracting the unstimulated pool concentrations from their counterpart anti-CD3– and CMV peptide–stimulated wells. Lactate data were analyzed in a blinded fashion. Positive wells were those with lactate concentrations ≥2 SD above the mean lactate concentration in the unstimulated wells. The lactate assay was set up in parallel with an IFN-γ FluoroSpot assay, using the same PBMCs and peptide pools. The FluoroSpot assay was processed at day 2, as previously described (13). In brief, the method involved incubating 2 × 10^5^ PBMCs in precoated FluoroSpot plates (Mabtech AB) in triplicate with gB or IE1 protein mix peptides (at a final concentration of 2 μg/ml per peptide) or unstimulated, or in the presence of anti-CD3 stimulation. The cells and medium were decanted from the plate, and the assay was developed following the manufacturer’s instructions. Developed plates were read using an AID iSpot reader (Oxford Biosystems) and counted using AID FluoroSpot V7 software (Autoimmun Diagnostika). All data were corrected for background cytokine production. The positive response cutoff was taken as a mean of 100 spot forming units per million, as previously determined in a larger study (13).

### Statistical analysis

All flow cytometry data were analyzed in-house with FlowJo (v10.1); all other data analyses and statistical fitting were performed in-house with Matlab (MathWorks) or GraphPad (Prism). Where appropriate, ANOVA and two-tailed *t* tests were performed to assess for differences between lactate concentrations in cell well cultures. Coefficient of variation analysis was performed on freeze–thaw samples. To assess for correlation between techniques in the suppression assay experiments, a least squares linear fit was performed. Sensitivity and specificity calculations were performed in the peptide-response experiments, using serology as the gold standard measurement technique.

## Results

### Activated CD4^+^ and CD8^+^ effector cells produce lactate in normoxic conditions

**FIGURE 1.**
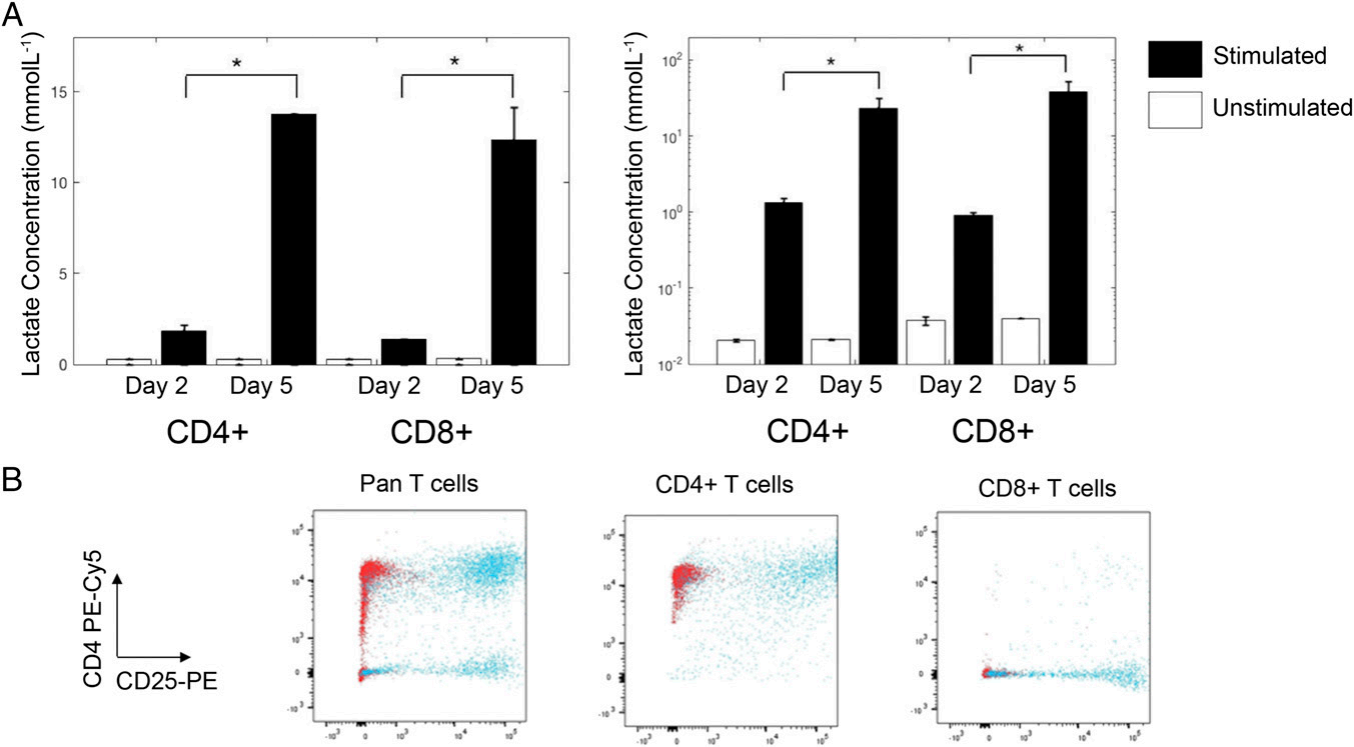
Extracellular lactate production by human T cells. (**A**) CD4^+^ and CD8^+^ T cells from two donors were cultured or not for 2 and 5 d with anti-CD3/CD28 Dynabeads, and supernatants were collected in triplicate for analysis of extracellular lactate. Lactate was normalized to viable cell number at each time point. (**B**) On day 5, the remaining cells were analyzed by flow cytometry for purity and activation status, as shown by CD4–PE–Cy5 versus CD25-PE staining. Red dots represent unstimulated cells, and blue dots represent stimulated cells. Unstimulated cell lactate output showed no change between days 2 and 5. *p < 0.01.

By day 2, the concentration of lactate measured in supernatant derived from CD3/CD28-stimulated CD4^+^ and CD8^+^ cells cultured in standard laboratory conditions (normoxia at 37°C with 5% CO_2_) was significantly higher than that derived from unstimulated cells (0.3 ± 0.1 versus 1.9 ± 0.3 and 0.4 ± 0.1 versus 1.46 ± 0.03 mmol/l, respectively, *p* < 0.01). Between days 2 and 5, lactate continued to accumulate in the supernatant of stimulated CD4^+^ and CD8^+^ cells (0.4 ± 0.2 versus 14.16 ± 1.82, 0.4 ± 0.1 versus 13.7 ± 0.4 mmol/l, *p* < 0.01). Correcting this for the number of live CD4^+^ and CD8^+^ cells at the end of culture revealed an increase in the amount of lactate produced per cell (Fig. 1, *p* < 0.01 in all cases). Lactate did not accumulate in the supernatant of unstimulated cells. Flow cytometric analysis confirmed purity and activation status of the cells (Fig. 1B).

### Extracellular lactate correlates well with T cell proliferation

**FIGURE 2.**
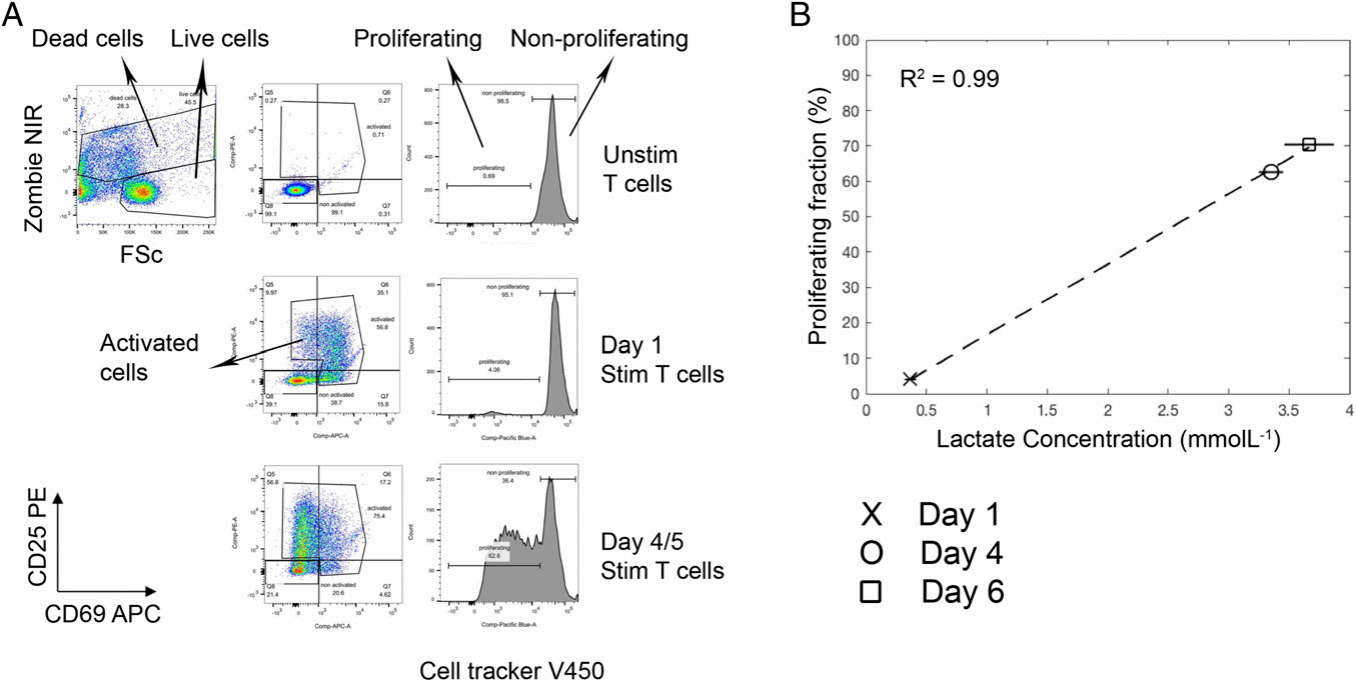
Increased total extracellular lactate correlates with increasing proliferation in stimulated T cell cultures. Cell proliferation dye V450–labeled T cells were cultured in the presence and absence of anti-CD3/CD28 Dynabeads for 6 d. At days 1, 4, and 6, cell culture supernatants were sampled for lactate analysis, and cells were analyzed by flow cytometry for proliferation status (cell proliferation dye dilution), as well as counterstained with dead cell marker (Zombie NIR) and Abs to CD69-allophycocyanin and CD25-PE. (**A**) The flow gating strategy: dead cells, activated cells, proliferating cells, and nonproliferating cells were gated. (**B**) Correlation of total extracellular lactate concentration with T cell proliferation.

Comparison of the temporal changes in total lactate obtained from the supernatants of CD3/CD28-stimulated T cells after day 1, 4, and 6 with flow cytometry data from the same culture well revealed a very strong correlation between T cell proliferation (defined by cell proliferation dye dilution) and total lactate (*R*^2^ = +0.99, Fig. 2). Cultures of activated T cells, expressing the early activation marker CD69 and/or CD25 but not yet proliferating, did not contain high levels of lactate, indicating that cells undergoing cellular proliferation use glycolysis to a greater extent than do nondividing activated T cells. Extracellular lactate concentration was unaffected by cell death (Supplemental Fig. 1).

### The sensitivity of lactate as a measure of T cell proliferation is comparable to thymidine at later time points

**FIGURE 3.**
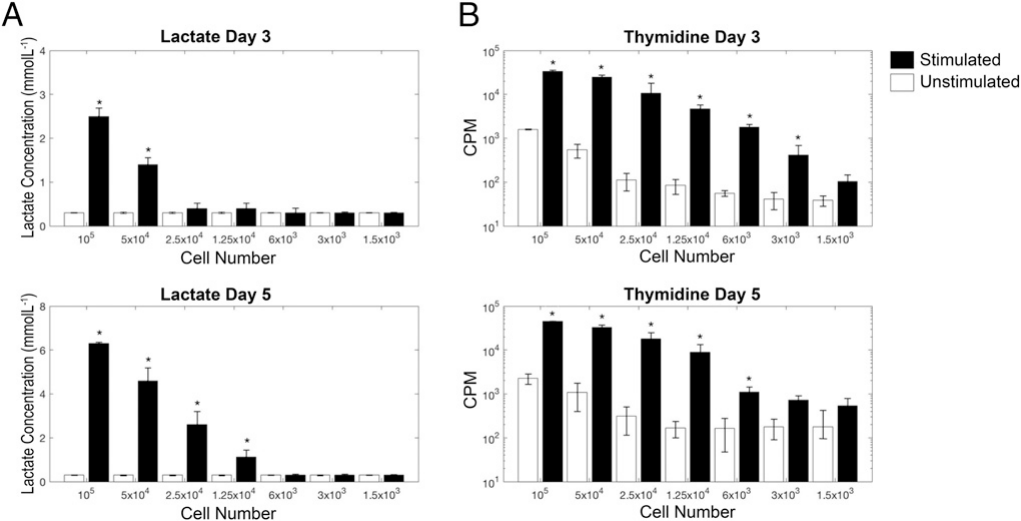
Comparison of the extracellular lactate proliferation assay with the thymidine-incorporation proliferation assay. Whole PBMCs were stimulated, in triplicate, with plate-bound anti-CD3/soluble anti-CD28, and supernatants were collected for lactate analysis prior to pulsing cells with thymidine. To test for sensitivity of the assay, cell numbers were titrated from 10^5^ cells per well to 500 cells per well. (**A**) Total lactate at days 3 and 5. (**B**) Incorporated thymidine (cpm) at days 3 and 5. Lactate data are shown on a linear scale, and thymidine data are shown on a log scale. *p < 0.05 versus unstimulated control.

Next, we went on to compare the sensitivity of extracellular lactate as a measure of T cell proliferation with the commonly used technique of [^3^H]thymidine incorporation, by culturing between 10^5^ and 1.5 × 10^3^ whole PBMCs with plate-bound anti-CD3 and soluble anti-CD28 for 3 and 5 d. Thymidine was able to discriminate between unstimulated and stimulated wells at a lower limit of 6 × 10^3^ PBMCs at days 3 and 5 (*p* < 0.01), although, at both time points, 1.25 × 10^4^ PBMCs was the lowest count that was significantly above the commonly accepted median cpm cutoff of 1000 (14). Lactate performed less well at day 3 (lower limit of sensitivity 5 × 10^4^ PBMCs, *p* < 0.01); however, at day 5, its sensitivity was comparable to that of thymidine (lower limit of sensitivity 1.25 × 10^4^, *p* < 0.01). Because this experiment was undertaken by stimulating T cells within whole PBMC cultures, the number of proliferating T cells that can be detected by both methods will be significantly less than 1.25 × 10^4^. Results from the sensitivity assay are shown in Fig. 3.

### Lactate is stable over multiple freeze–thaw cycles

**FIGURE 4.**
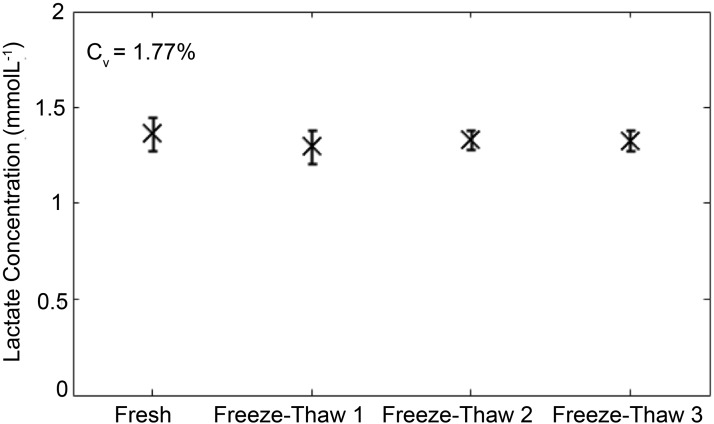
Stability of lactate measurements. Extracellular lactate is stable over several freeze–thaw cycles. A total of 3 × 10^6^ Pan T cells was stimulated in a 24-well plate with anti-CD3/CD28 Dynabeads for 5 d, and 1 ml of supernatant was collected for analysis. Total lactate concentration is shown for the same cell culture supernatant that was repeatedly freeze–thawed.

To determine the stability of lactate after freeze–thawing, 10^6^ T cells were stimulated with anti-CD3/CD28 Dynabeads for 5 d, and the concentration of lactate in aliquots subjected to one to three rounds of −20°C freeze–thawing was compared with the concentration of lactate measured in fresh supernatant. Analysis of the variance in results revealed highly reproducible results over a course of three freeze–thaw cycles, with a coefficient of variation of 1.77% over four test and retest cycles (Fig. 4).

### Extracellular lactate is as accurate as cell proliferation dye in determining Treg suppression

**FIGURE 5.**
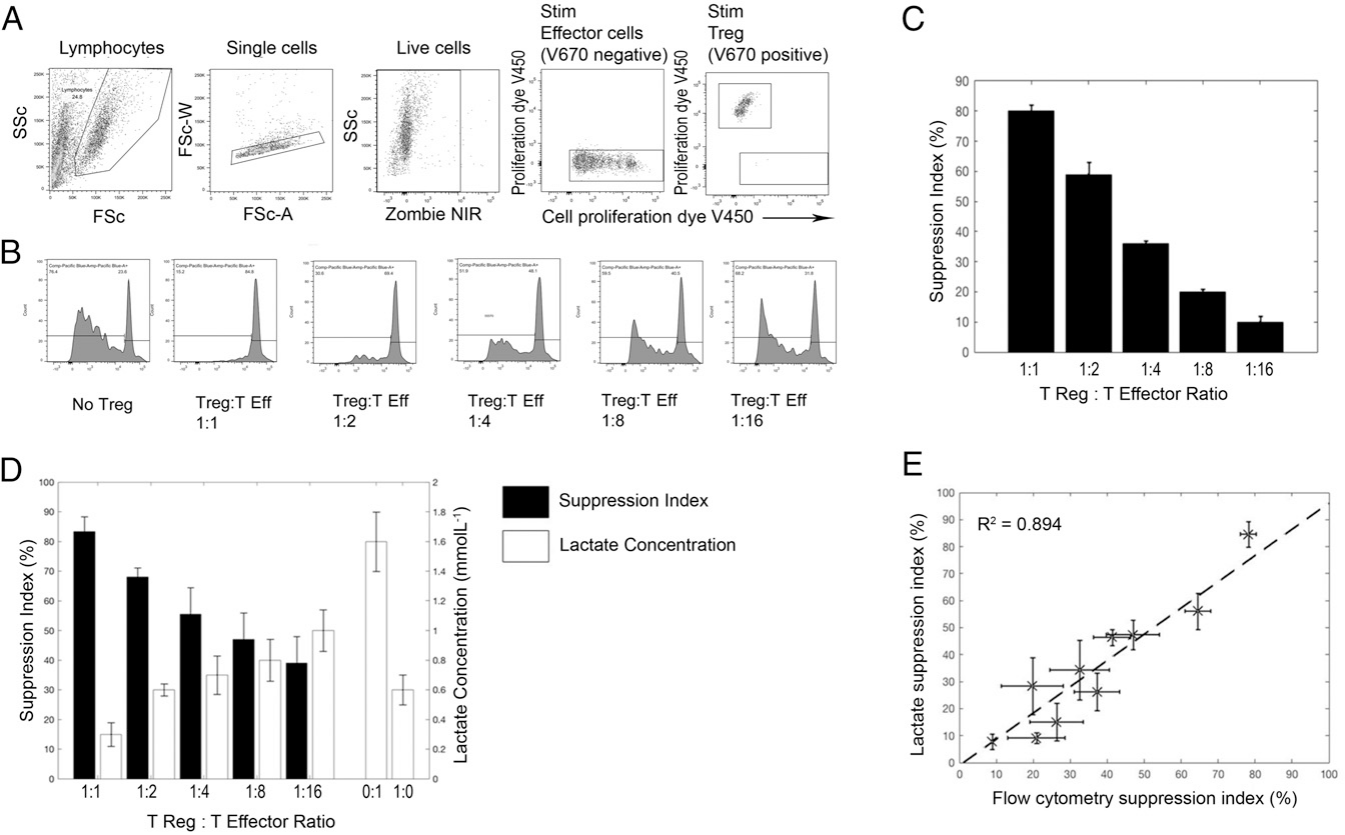
Use of lactate in analyzing a Treg-suppression assay. Treg-suppression assays were performed by coculturing V450 cell proliferation dye–labeled CD4^+^ effector T cells with V670 cell proliferation–labeled CD4^+^CD25^hi^CD127^low^ Tregs at a 1:1 ratio and doubling dilutions thereafter, in triplicate. (**A**) Flow gating strategy of this assay. Live V670^−^ cells (non-Treg) were taken as effector cells. (**B**) V450 proliferation dye dilution of the effector T cells [gated in (A)], with and without Tregs. (**C**) Percentage suppression of the effector T cell response in the presence of Tregs was calculated (according to the given equation; see *Materials and Methods*); suppression by Tregs was titratable. (**D**) Amount of lactate produced by 10^4^ effectors (0:1), 1 × 10^4^ Tregs (1:0), and 10^4^ effectors cultured with various numbers of Tregs, corrected for the amount of lactate in the 1:0 well. The black bars represent the calculated lactate suppression index. (**E**) Combined data from three suppression assays, correlating the suppression index calculated by flow cytometry with that calculated by lactate.

In keeping with the literature (10), under normoxic conditions in vitro, Treg lactate production was relatively low (compared with Teffs) and did not increase following activation (Supplementary Fig. 2). Therefore, we went on to explore the usefulness of measuring lactate in a classical Treg-suppression assay in which the coculture would not be complicated by the increased background production of metabolites from the regulatory cell pool. Cell proliferation dye dilution was used as the comparator. A representative flow cytometric analysis is shown in Fig. 5A–C and a lactate analysis is shown in Fig. 5D. As expected, a titratable effect of modifying the Treg/Teff ratio was observed using both methods (Fig. 5C, 5D). Furthermore, calculation and comparison of the suppression index obtained over multiple suppression assays revealed a strong correlation between estimates of suppression obtained from lactate and those obtained by flow cytometry (see Eqs. 1, 2, *R*^2^ = +0.894).

### Extracellular lactate provides high specificity and sensitivity in predicting CMV status

Given the ease with which lactate can be measured, as well as its sensitivity, stability, and low cost, we went on to test its usefulness in interrogating T cell responses to libraries of overlapping peptides, a relatively high-throughput technique commonly used to assess vaccine-induced or other immunological responses. In brief, cryopreserved PBMCs from 10 healthy individuals (5 known to be CMV seropositive and 5 seronegative) were cultured with IE1 and gB protein CMV peptide pools; both proteins have been shown to trigger T cell responses in many donors (15). Supernatant lactate production was compared with IFN-γ production, as measured by FluoroSpot. CMV serology was taken as the “gold standard”; CD3 stimulation was included as a positive control. Two donors were excluded from the analysis owing to a poor anti-CD3 response in both the lactate and FluoroSpot assays, possibly as a result of using cryopreserved PBMCs that did not recover well. The remaining eight donors produced a positive anti-CD3 response and, therefore, were included in the analysis. Of note, donor 1 produced very little lactate in response to CD3 stimulation (just above background), whereas their cells made significant amounts of IFN-γ, highlighting the recognized difference between proliferation (which we have shown correlates strongly with lactate production) and cytokine secretion (16).

**FIGURE 6.**
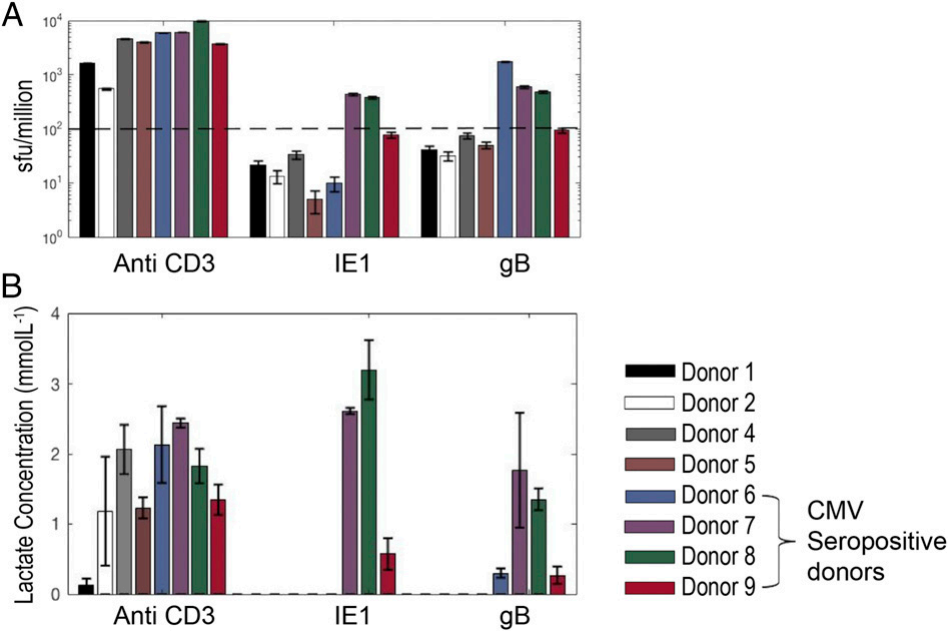
Screening of blood donors for responses to CMV peptides. Data from PBMCs of eight healthy donors, cultured with soluble anti-CD3 or one of two different CMV peptide pools (IE1 and gB; 1 μg/ml). PBMCs were plated at 10^5^ cells per well in triplicates in 96-well plates. (**A**) IFN-γ ELISPOT was performed at 48 h, and a mean spot-forming unit per million > 100 was taken as a positive response. Extracellular lactate was measured in cell culture supernatants at days 2 and 5. (**B**) Day-5 positive total lactate concentrations. All four seropositive donors were identified as responders to one or both peptides by both methods.

Two independent blinded analysts concluded that donors 6, 7, 8, and 9 were CMV^+^ from the lactate results; this was in 100% agreement with the serology and FluoroSpot results after unblinding (Fig. 6). Further analysis of the use of lactate production as a predictor for CMV status revealed 100% specificity and sensitivity of the technique in comparison with serology and FluoroSpot.

## Discussion

By making use of what is known about the reliance of activated Teffs on Warburg metabolism, we have demonstrated that extracellular lactate can be used to accurately determine T cell proliferation in vitro, with usefulness across a range of assays.

In this study, we used spectrophotometry to quantify lactate via the lactate-to-pyruvate exchange reaction catalyzed by lactate dehydrogenase. This represents a simple, inexpensive, and widely available approach to assess T cell activation. Cell culture supernatants can be harvested and stored at −20°C until the researcher is ready to process them. There are no additional time-consuming steps to be performed, and, unlike thymidine, there is no need for radioactive and potentially carcinogenic material to be handled. The stability of lactate over multiple freeze–thaw cycles also opens up the possibility that this method could be used to measure T cell proliferation post hoc, in stored supernatants collected for other purposes, such as the measurement of cytokines. Commercial lactate measurement kits are also available as simple-to-use colorimetric assay kits. These assays were originally designed for the analysis of tumor cells in culture; however, we suggest that they could be repurposed to effectively measure T cell metabolism and proliferation.

In contrast to thymidine, lactate is not a “snapshot” measure of cell proliferation at a single time point; rather, it is an endpoint/cumulative measure that provides information regarding the replicative history of the cultured cells. In that regard, it is more akin to cell proliferation dyes. As a result, lactate is less sensitive than thymidine at early time points; however, at later time points, its sensitivity is comparable.

Several other nonradioactive single time point–methods exist for analyzing T cell proliferation, such as the MTT assay, which is a metabolism-based colorimetric assay. However, this suffers from a relative lack of sensitivity and has issues with toxicity. An MTT assay requires cells to be pulsed with substrate; therefore, multiplexing this assay with other methods is not possible. In contrast, lactate accumulates naturally throughout the period of cell culture, with no need for additional cytotoxic reagents to be added, so it can be readily used alongside other assays (e.g., cytokine analysis or flow cytometry). Through costaining with Abs against additional cellular markers, flow cytometric methods enable significant additional details about the nature of the cellular response to be interrogated; however, flow cytometry is labor intensive and relatively costly. Furthermore, the level of detailed information that flow cytometry can provide is not always required.

We have demonstrated that, under standard laboratory normoxic conditions, extracellular lactate strongly correlates with T cell proliferation (*R*^2^ = +0.99). We assume that this would not be the case if cells were to be cultured in hypoxic conditions, in which proliferating and nonproliferating cells would use glycolysis to satisfy their energy needs (17).

Although the literature on Treg metabolism is complex, and recent studies have demonstrated that the in vivo environment may influence their metabolic requirements, it is generally accepted that in vitro FOXP3^+^ Tregs rely more on fatty acid oxidation and do not undergo glycolysis. In addition, it has recently been suggested that FOXP3, the key transcription factor in Tregs, actively turns off glycolysis (10, 17–19). In keeping with this, we have shown that, in normoxic conditions, in vitro–cultured Tregs produce relatively little lactate and that production does not increase with activation. Given this, we reasoned that lactate may be a particularly attractive method for assessing Treg suppression of Teffs, because the cocultures would not be complicated by significant background production of metabolites from the regulatory cell pool. Indeed, this was demonstrated to be the case, with suppression of lactate production showing strong correlation with suppression of T cell proliferation, as determined by cell proliferation dye and flow cytometry (*R*^2^ = +0.894). As described in *Materials and Methods*, the amount of lactate produced by Teffs in the presence of Tregs was corrected by the amount of lactate produced by 10^4^ stimulated Tregs (regardless of the actual number of Tregs in any given well), raising the possibility that our method may overestimate suppression at the lower Treg/Teff ratios. However, this effect was found to be small and was not statistically significant. Given its simplicity, sensitivity, stability, and cost effectiveness, we have shown that lactate could be useful in interrogating T cell responses in high-throughput assays, such as measuring responses to libraries of overlapping peptides to assess vaccine-induced or other immunological responses. To assess this, we examined its usefulness in identifying CMV-seropositive healthy individuals and found it to be 100% sensitive and specific in the sample tested. A summary of the techniques used in this article, including their cost and time required for analysis, is shown in Table I.

**Table I.** Summary of cell culture analysis techniques

Name	Type of Measurement	Approximate Cost per Well ($)	Radiation Exposure	Experimental Time	Experimental Steps
Lactate	Cumulative	1	No	Approximately 30 min	Removal of cell culture supernatants Spectrophotometry
Flow cytometry	Cumulative	6	No	Several hours	Cell proliferation dye labeling during assay set-up
					Harvesting and counterstaining of cells with Abs
					Flow cytometry acquisition and analysis
Thymidine	Snapshot	1	Yes	>12 h	Pulsing of cells with thymidine (usually overnight)
					Cell harvesting

In conclusion, we believe that the measurement of extracellular lactate is a safe, reliable, sensitive, fully quantifiable, and cost-effective method for analyzing T cell proliferative responses in vitro that can complement, and in selected circumstances replace, more traditional methods. In particular, we suggest that lactate may prove to be particularly useful in high-throughput screening assays.

## Supplementary Material

Data Supplement
