# Supplementary material for: Extracellular Lactate: A Novel Measure of T Cell Proliferation
Source: J Immunol. 2017 Dec 29;200(3):1220–6. doi: 10.4049/jimmunol.1700886 (PMC5776880; doi:10.4049/jimmunol.1700886)
Supplement: Data Supplement [file JI_1700886.zip › JI_1700886_Supplemental_Figures_1.pdf]

## Supplementary Figure 1

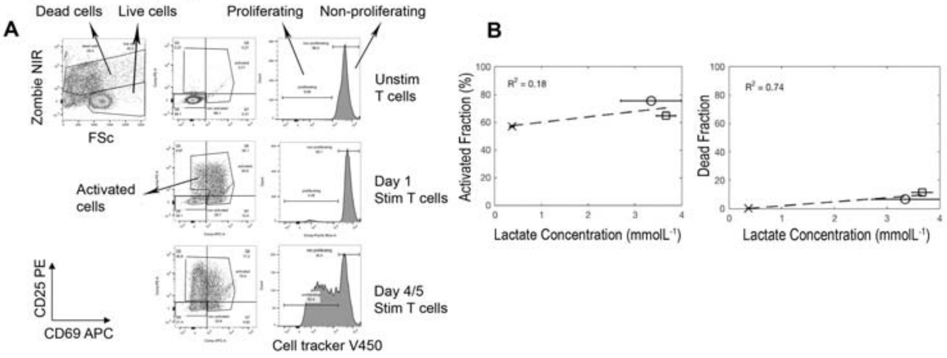


**Increased total extracellular lactate does not correlate with activated, non-proliferating fraction, nor with dead fraction.**
Cell proliferation tracker V450 labelled T cells were cultured in the presence and absence of anti-CD3/28 dynabeads for 6 days. At days 1, 4 and 6, cell culture supernatants were taken for lactate analysis and cells were analysed by flow cytometry for activation status (cell proliferation low, high CD69-APC and CD25-PE) as well as counterstained with dead cell marker (zombie NIR). Flow was analysed on a BD Canto II and in flowjo version 10. The flow gating strategy is shown in A: dead cells, activated cells, proliferating and non-proliferating cells were gated. The correlation of total extracellular lactate concentration with activated, non-proliferating (left plot) and dead cells (right plot) is shown in B (correlations not statistically significant, $p = 0.2$ and $p = 0.375$, respectively).

## Supplementary Figure 2

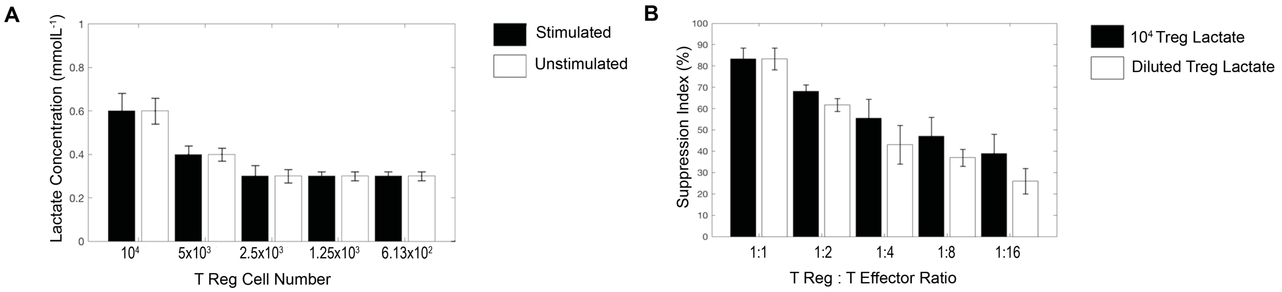


**Lactate does not increase with Treg stimulation and the suppression index (normally calculated by correcting for the amount of lactate produced by $10^4$ Tregs) is not significantly altered by correcting for the amount of lactate produced by the exact number of Tregs in any given well.**
A: $10^4$ T Regs, with subsequent dilutions, were cultured in RPMI with and without anti CD3/28. Supernatants were analysed at day 6, showing a small decrease in lactate with cell dilution. B: Shows the suppression index calculated by correcting for the amount of lactate produced by $10^4$ Tregs (white bars) vs. correcting for the amount of lactate produced by the exact number of Tregs at any given dilution (black bars). No significant difference was seen
